# Malaria in pregnancy in rural Gabon: a cross-sectional survey on the impact of seasonality in high-risk groups

**DOI:** 10.1186/1475-2875-12-412

**Published:** 2013-11-13

**Authors:** Mario J Jäckle, Christian G Blumentrath, Rella M Zoleko, Daisy Akerey-Diop, Jean-Rodolphe Mackanga, Ayôla A Adegnika, Bertrand Lell, Pierre-Blaise Matsiegui, Peter G Kremsner, Ghyslain Mombo-Ngoma, Michael Ramharter

**Affiliations:** 1 Centre de Recherches Médicales de Lambaréné, Lambaréné, Gabon; 2 Ngounié Medical Research Centre, Fougamou, Gabon; 3 Institut für Tropenmedizin, Universität Tübingen, Tübingen, Germany; 4 Department of Medicine I, Division of Infectious Diseases and Tropical Medicine, Medical University of Vienna, Vienna, Austria

**Keywords:** Malaria, Pregnancy, Risk factors, Parity, Age, Gestational age, Season, Seasonality, IPTp

## Abstract

**Background:**

Malaria remains one of the most important infectious diseases in pregnancy in sub-Saharan Africa. Whereas seasonal malaria chemoprevention is advocated as public health intervention for children in certain areas of highly seasonal malaria transmission, the impact of seasonality on malaria in pregnancy has not yet been investigated for stable, hyper-endemic transmission settings of Equatorial Africa. The aim of this study was to investigate the influence of seasonality on the prevalence of malaria in pregnancy in Gabon.

**Methods:**

The study was conducted at a rural district hospital in Gabon between January 2008 and December 2011. At first antenatal care visits demographic data, parity, age, and gestational age of pregnant women were documented and thick blood smears were performed for the diagnosis of malaria. Seasonality and established risk factors were evaluated in univariate and multivariate analysis for their association with *Plasmodium falciparum* infection.

**Results:**

1,661 pregnant women were enrolled in this study. Participants presenting during high transmission seasons were at significantly higher risk for *P. falciparum* infection compared to low transmission seasons (adjusted odds ratio [AOR] 1.91, 95% confidence interval [CI] 1.39-2.63, *p* < 0.001). Established risk factors including parity (AOR 0.45, CI 0.30-0.69, *p* < 0.001 for multipara versus paucipara) and age (AOR, CI and p-value for women aged 13–17, 18–22, 23–27 and ≥28 years, respectively: AOR 0.59, CI 0.40-0.88; AOR 0.57, CI 0.34-0.97; AOR 0.51, CI 0.29-0.91) were significant risk factors for *P. falciparum* infection. High-risk groups including nulli- and primipara and younger women aged 13–17 years showed a disproportionately increased risk for malaria in high transmission seasons from 17% to 64% prevalence in low and high transmission periods, respectively.

**Conclusion:**

Seasonal variations lead to important differences in the risk for *P. falciparum* infection in pregnancy in the setting of central African regions with stable and hyper-endemic malaria transmission. The seasonal increase in malaria in pregnancy is most pronounced in high-risk groups constituted by young and pauciparous women. The evaluation of tailored seasonal prevention strategies for these high-risk populations may, therefore, be warranted.

## Background

An estimated 25 million women are at risk to become infected by *Plasmodium falciparum* during pregnancy in sub-Saharan Africa each year [1,2]. Adverse birth outcomes due to malaria in pregnancy include an increased prevalence of maternal anaemia, low birth weight, premature birth and neonatal mortality [3,4]. The World Health Organization (WHO) recommends a three-pronged strategy against malaria in pregnancy based on the use of insecticide-treated bed nets (ITNs), effective case management and the administration of intermittent preventive treatment against malaria in pregnancy (IPTp) with sulphadoxine-pyrimethamine (SP) [5,6].

Malaria epidemiology in Gabon is characterized by stable, hyper-endemic transmission of more than 90% *P. falciparum*[7]. In previous surveys little variation in malaria transmission throughout the year has been observed [7]. The climate is characterized by two rainy and two dry seasons [8]. Malaria in pregnancy is among the most important health problems for pregnant women and the national malaria control programme recommends the use of ITNs and SP-IPTp. The implementation of IPTp has led to a considerable decline in *P. falciparum* infection in pregnant women in Gabon [9]. Established risk factors for adverse birth outcome and malaria in pregnancy including pauciparity and young age have been equally established in Gabon [10], however there is no information about the influence of seasonal variations in malaria transmission on the prevalence of malaria in pregnancy in Gabon.

Based on the observation of high seasonality of malaria transmission in certain African regions including large parts of the Sahel, the concept of seasonal malaria chemoprevention has been developed for children [11]. This concept advocates administration of amodiaquine (AQ) and SP only during peak transmission seasons to minimize drug exposure and at the same time maximizing the protective effect of AQ and SP against malaria. To date it is unclear whether similar interventions would be feasible for the prevention of malaria in pregnancy. To better understand the impact of seasonality on the risk for malaria in pregnancy in a central African region with little seasonal variation in malaria transmission we conducted this cross sectional study.

## Methods

### Study region and study population

This study was conducted in the town of Fougamou (approximately 8,000 inhabitants) in the rural province of la Ngounié in Gabon. The region is located in a hilly, rainforest covered area with predominance of subsistence farming as professional activity. The climate is equatorial and meteorological data indicate two dry seasons (December - January and June - September) and two rainy seasons (September - November and February – May, [8,12]. The average annual temperature is 26°C and humidity is above 80% [8]. Malaria transmission is characterized as hyperendemic and *P. falciparum* shows high resistance against chloroquine and increasing resistance against SP [13,14]. The Ngounié Medical Research Centre, a satellite site of the Centre de Recherches Médicales de Lambaréné [15], is providing laboratory and parasitological services for the local governmental hospital, which is the only health care institution serving the local population.

Pregnant woman presenting for their first antenatal care visit at the hospital were enrolled in this study. All pregnant women received a mother-child health booklet at presentation and bodyweight, blood pressure, uterine height and gestational age according to the first day of the last menstruation were recorded. All women were offered thick blood smear evaluation performed according to the Lambaréné method [16] for the diagnosis of malaria which were read and prove-read by experienced study professionals guaranteeing high quality data. The following data were captured in a central register: age, parity, date of visit and results of malaria diagnostics. Data were extracted from this register and entered in an electronic database. All women with malarial infection received treatment following current national recommendations.

All thick blood smears were defined as positive if any asexual forms of *P. falciparum* were observed. Pregnant women were categorized as nulli-, primi- and multipara depending on the number of self-reported previous pregnancies. Women were categorized in the following four age groups based on legal age and 5 year intervals: 13–17 years, 18–22 years, 23–27 years, and 28 or more years of age. With the aim to determine low- and high-risk seasons for malaria transmission throughout the year, the proportion of positive blood smears was calculated for each month during the study period. The month March (malaria prevalence of 13.9%) was chosen as cut-off point determining the risk-periods based on concordant annual trends during the study period: Low-risk periods were in April and between June and September (high-risk periods were May and October - March). Gestational age was calculated according to the reported first day of last menstruation. Ethical approval for this retrospective analysis of clinical data that were obtained from routine clinical records was provided by the Comité d’Ethique Régional et Independent de Lambaréné.

### Statistical analysis

Data were imported for further analysis into a commercial statistical software package (IBM SPSS Statistics, version 19.0). Descriptive analyses were performed and a binary logistic regression model was used to compute the adjusted odds ratios (AOR) of independent risk factors for malaria in pregnancy. To minimize exclusion of data due to missing values binary logistic regression model was computed for two scenarios: including 1) participants with all data (parity-, age-, season- and trimester-group) available for analysis and 2) participants with data for parity, age and season. Since this sensitivity analysis demonstrated only minimal differences, the model with fewest exclusion is shown. Statistical significance was defined by a likelihood ratio with a p-value below 0.05. 95% confidence intervals (CI) were calculated by the exact binominal method.

## Results

### Characteristics of study population

Between January 2008 and December 2011 a total of 1,661 pregnant women attended the hospital for their first antenatal care visit. Table 1 shows the demographic characteristics of the participating women. The total number of positive blood smears was 263 (16%) during the study period and proportions of *P. falciparum* infections diagnosed at first antenatal care visit are also given in Table 1. Mean parasitaemia of all positive blood smears was 1075 per microlitre blood. The distribution of risk factors (parity, gestational and maternal age) among the study population did not vary depending on the month of presentation during the study-period.

**Table 1:** Demographic characteristics of study population and prevalence of malaria in pregnancy in 2008–2011

**Demographic characteristics**			
*n* (number)		1661	
Age* (in years)		23 (17–34;1634)	
Age groups stratified			
(13–17, 18–22, 23–27, 28+; *n*)		17%, 32%, 22%, 29%; 1634	
Parity*		1 (0–5;1545)	
Parity stratified		29%, 22%, 49%; 1545	
(NP, PP, MP; *n*)**			
Parity* in age group 1		0 (0–1; 264)	
Parity* in age group 2		1 (0–2; 491)	
Parity* in age group 3		2 (1–4; 341)	
Parity* in age group 4		4 (2–7; 428)	
Nullipara* (Age in years)		18 (15–34; 448)	
Primipara* (Age in years)		20 (18–26; 333)	
Multipara* (Age in years)		28 (22–38; 743)	
Trimester***		24%, 69%, 8%; 769	
(1st, 2nd, 3rd; *n*)			
**Malaria prevalence**			
**Year**	***n***	**Malaria pos. n (%)**	**95% CI**

2008	325	43 (13%)	10–17%
2009	391	54 (14%)	11–18%
2010	486	93 (19%)	16–23%
2011	444	73 (16%)	13–20%
2008–2011	1646	263 (16%)	14–18%

Differences between the total number of enrolled women and the total number in specific groups are due to missing data for the particular variable.

*Median (10–90% quantiles; *n*).

**NP, Nullipara; PP, Primipara; MP, Multipara.

***Percentage of sample.

### Risk factor analysis for malaria in pregnancy

Parity, age and season were significantly associated with the risk for malaria computed by the binary logistic regression (Table 2). Multipara were significantly less likely to be diagnosed with malaria compared to nullipara and primipara. When combining nulli- and primipara in one group there was a statistically significant difference compared to multipara in the prevalence of malaria (AOR 0.45, CI 0.30-0.69, *p* < 0.001, not shown in Table 2).

**Table 2:** Results of the binary logistic regression: malaria prevalence in different risk groups (2008–2011)

		***n***	**Malaria pos. *n *** **(%)**	OR* (95% CI)	AOR** (95% CI)	*p*-value**
Parity	Nullipara	442	114 (26%)	1.00	1.00	Ref
	Primipara	330	59 (18%)	0.63 (0.44–0.89)	0.79 (0.53–1.17)	0.231
	Multipara	738	66 (9%)	0.28 (0.20–0.39)	0.39 (0.24–0.64)	< 0.001
Age in years	13–17	262	77 (29%)	1.00	1.00	Ref
	18–22	484	83 (17%)	0.50 (0.35–0.71)	0.59 (0.40–0.88)	0.010
	23–27	339	40 (12%)	0.32 (0.21–0.49)	0.57 (0.34–0.97)	0.038
	28+	425	39 (9.2%)	0.24 (0.16–0.37)	0.51 (0.29–0.91)	0.023
Season	Low-risk	547	61 (11%)	1.00	1.00	Ref
	High-risk	963	178 (19%)	1.81 (1.32–2.47)	1.91 (1.39–2.63)	< 0.001
Trimester	1	163	36 (22%)	1.00	1.00	Ref
	2	496	76 (15%)	0.64 (0.41–0.99)	0.68 (0.43–1.08)	0.105
	3	57	6 (11%)	0.42 (0.16–1.04)	0.42 (0.16–1.08)	0.070

*OR (odds ratio).

**AOR (adjusted odds ratio and *p*-value, calculated by the binary logistic regression model).

Malaria prevalence decreased significantly from age group 1 to age group 4 and pregnant women attending antenatal care visits in their first trimester had the highest risk for *P. falciparum* infection with a prevalence of 23% (*n* = 40). In binary logistic regression for gestational age defined as an ordinal variable, the model showed a significant association of gestational age with a decrease in malaria positivity from the first to the third trimester (AOR 0.66, CI 0.45-0.97, *p* = 0.035, not shown in Table 2).

### Impact of seasonality on the risk for malaria in pregnancy

**Figure 1:**
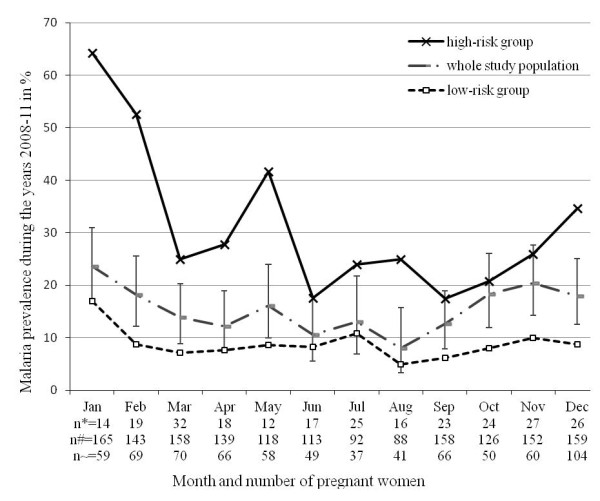
**Seasonal prevalence of malaria in pregnancy of high-risk versus low-risk groups during the study period 2008–2011.** The malaria prevalence is aggregated by month for all the years 2008 until 2011. The solid curve (drawn in black) is a high-risk group composed of nulli-, primipara pregnant women and age group 1. The dashed curve (drawn in grey) includes the whole study population, 95% confidence intervals are given as error bars. The dashed curve (drawn in black) is a low-risk group composed of multipara and age group 2–4. The number of pregnant women by month is given for all groups (n* = high-risk group, *n*# = whole study population, *n* ~ = low-risk group).

The dashed curve (drawn in grey) in Figure 1 shows the distribution of the malaria prevalence in pregnancy aggregated by month of presentation for the first antenatal care visit for the study period 2008–2011. Monthly proportions for *P. falciparum* infection ranged from 8% (*n* = 7) in August to 24% (*n* = 39) in January. In the course of the year malaria positivity declined from January to April to 12% (*n* = 17), increased thereafter to 16% (*n* = 19) in May for a further decline to 11% (*n* = 12) in June. Malaria prevalence increased again between September and November to 20% (*n* = 31). This pattern was comparable for all years during the study period. The variation in the prevalence of malaria in pregnancy depending on the annual season was statistically significant as assessed in logistic regression analysis (high- versus low-risk season: AOR 1.91, CI 1.39-2.63, *p* < 0.001).

### Impact of seasonality on the risk for malaria in pregnancy in high-risk groups

The solid curve and the dashed curve (drawn in black) in Figure 1 depicts the distribution of malaria prevalence in pregnancy from 2008–2011 for high-risk pregnant women including young (13–17 years of age), nulli- and primiparous women compared to low-risk women (age above 17 years, at least two pregnancies). In every month the high-risk group (solid curve) had a markedly higher prevalence of *P. falciparum* infection than the low-risk group (dashed curve). Highest prevalence was observed in January (64%) and lowest in September (17%), indicating a higher degree of variation in this high-risk group compared to the general study population.

## Discussion

This study demonstrates that marked seasonal variations in the prevalence of *P. falciparum* infection in pregnancy occurs in a rural Central African community in Gabon. The finding of an AOR of 1.91 seems surprisingly high on the background of perennial, stable and hyper-endemic malarial transmission in Gabon [7]. Besides seasonality several other risk factors including parity and maternal and gestational age were independently associated with the risk for *P. falciparum* infection. Whereas these data confirm previous findings [8,10,17-22], the sub-analysis of a high-risk group of young and pauciparous women showed even more pronounced seasonal variations in malaria prevalence with maximum monthly prevalence rates varying from 64% in January to 17% in September. This potentiating effect of seasonality on established risk factors in the setting of perennial stable malaria transmission has not yet been well described. The epidemiological description of these sub-groups of pregnant women with disproportionally high-risk for *P. falciparum* infection may emphasize the need of effective prevention programs in this high-risk group for the prevention of malaria in pregnancy.

To date SP-IPTp is - besides the use of ITNs and case management - the recommended strategy of the WHO in most of sub-Saharan African countries to combat malaria in pregnancy [6]. Current recommendations advocate for the provision of at least three therapeutic doses of SP to all pregnant women after the first trimester [5]. The markedly increased risk for malaria in pregnancy in certain risk groups such as very young women, women in their first pregnancy and women having pregnancies during high transmission seasons is not reflected by the uniform recommendation for SP-IPTp in sub-Saharan Africa [5,6]. However, these data may be seen as supportive evidence justifying further clinical evaluation of tailored prevention strategies based on seasonal and demographic risk stratification.

Whereas our study aptly analyses risk factors and their association with malaria in pregnancy, several limitations of the study design need to be acknowledged. Firstly, this study is designed as a cross-sectional survey collecting data only at the first antenatal care visit. Therefore no robust estimate on the incidence of *P. falciparum* infections in the course of pregnancy can be drawn from this study. Similarly, data have been obtained from a hospital registry and potential problems in data accuracy can therefore not be excluded. Finally, the influence of concomitant intake of anti-malarial drugs cannot be ruled out. However, since all women presented for their first antenatal care visit, no major confounding by the intake of SP-IPTp is anticipated.

## Conclusion

Even in Central African regions with stable, perennial and hyper-endemic malaria transmission, seasonality leads to important variations in the prevalence of malaria in pregnancy. These variations are even more pronounced in high-risk groups of young and pauciparous women. These findings evoke reflexions on the potential usefulness of more individualized prevention strategies based on a risk-benefit analysis, for low- and high-risk groups among pregnant women in sub-Saharan Africa.

## Competing interests

The authors declare that they have no competing interests.

## Authors’ contributions

CGB, MJJ, RMZ and MR developed the idea for this paper. MJJ conceived the analyses and wrote the first draft of the manuscript. MJJ, CGB and MR drafted the final manuscript. BL conducted the binary logistic regression. All authors read and approved the final manuscript.
